# A BHR Composite Network-Based Visualization Method for Deformation Risk Level of Underground Space

**DOI:** 10.1371/journal.pone.0127088

**Published:** 2015-05-26

**Authors:** Wei Zheng, Xiaoya Zhang, Qi Lu

**Affiliations:** Key Laboratory for Optoelectronic Technology and System of the Education Ministry of China, College of Optoelectronic Engineering, Chongqing University, Chongqing, China; University of Connecticut, UNITED STATES

## Abstract

This study proposes a visualization processing method for the deformation risk level of underground space. The proposed method is based on a BP-Hopfield-RGB (BHR) composite network. Complex environmental factors are integrated in the BP neural network. Dynamic monitoring data are then automatically classified in the Hopfield network. The deformation risk level is combined with the RGB color space model and is displayed visually in real time, after which experiments are conducted with the use of an ultrasonic omnidirectional sensor device for structural deformation monitoring. The proposed method is also compared with some typical methods using a benchmark dataset. Results show that the BHR composite network visualizes the deformation monitoring process in real time and can dynamically indicate dangerous zones.

## Introduction

Safety accidents frequently occur in large-scale construction of underground space engineering [1, 2]. Safety monitoring of underground space is increasingly becoming a critical need for national economic development. However, monitoring data on underground space engineering involve large and complex features. Moreover, the existing visual tools are often based on specific measurement techniques. These techniques, such as photogrammetric, total station, and 3D laser scanning techniques, mainly focus on measurement data processing. In the process, such methods neglect the practicality of displaying risk levels. Thus, monitoring information on underground space safety and evaluating and forecasting its status are difficult, which in turn causes serious obstacles to underground space exploitation.

Some studies have been conducted on the visual technology of safety monitoring. For example, Smith and Brown have made some advances in directional borehole radar data analysis and visualization [3]. Tang has shown that the difference evolution arithmetic and visualization toolkit can be used to calculate and evaluate the state of tunnel surrounding rock [4]. A 3D laser scanning system has also been used to acquire and visualize monitoring data on underground space deformation [5, 6]. Chen has studied the browsing modes of the 3D simulation view in monitoring underground construction [7]. An approach has also been proposed for safety management of metro construction using 4D visualization technology [8]. Laser ultrasonic scanning excitation and integrated piezoelectric have been used in visualizing the defects in composite aircraft manufacturing and the damages of the debonding mode [9]. A wireless strain monitoring system, which integrates local tethered data acquisition and long-range wireless data transmission, has been developed for real-time strain monitoring and visualization of building safety [10]. Expanding on the original work in field construction by describing recent advances in both activity- and operation-level construction, Kamat et al. showed that graphical 3D visualization can serve as an effective communication method [11]. Moreover, bridge information modeling has become an effective tool in bridge engineering construction and visualization [12]. Continuous analytic techniques based on fracture mechanics and acoustic-emission analytics, along with software infrastructure, have been applied in real-time monitoring [13]. Glisic et al. researched and proposed the accessibility and visualization principles of heterogeneous monitoring data [14]. Other studies presented a general dynamic visualization model for SHM, which results in a dynamic and interactive visualization process [15]. A WSN monitoring framework based on 3D visualization [16] and a wireless data acquisition framework for structural health monitoring and control have also been presented [17].

Although various strategies for visualizing monitoring data have been developed, strategies for underground space safety remain a great challenge because of the following reasons: First, the underground space environment is highly complex, and numerous parameters affect safety monitoring [18, 19]. Most of the current methods are capable of handling only a limited number of parameters [20]. Second, the existing visualization techniques generally focus on a specific topography [21, 22]; research on the universal visualization model can still be expanded further. Third, the current methods have achieved mainly the visualization of monitoring data with specific physical characteristics, such as strain, temperature, or crack [23–25]. However, only simple methods, such as the threshold partition, are adopted for abstract risk-level characteristics. Given these limitations, an intelligent, dynamic, and real-time visualization technique is urgently needed. The present study proposes a visualization technique for underground space deformation risk level based on a BP-Hopfield-RGB (BHR) composite network. Through parallel inputting of multiple environmental parameters, the method constructs a universal model for monitoring underground space safety. Complex environmental factors are integrated by the BP neural network (BPNN), and dynamic monitoring data are automatically classified in the Hopfield network. Combined with the RGB color space, the deformation risk level is displayed in real time. Thus, the dangerous zones in underground space are indicated and located quickly.

## Method

**Fig 1:**
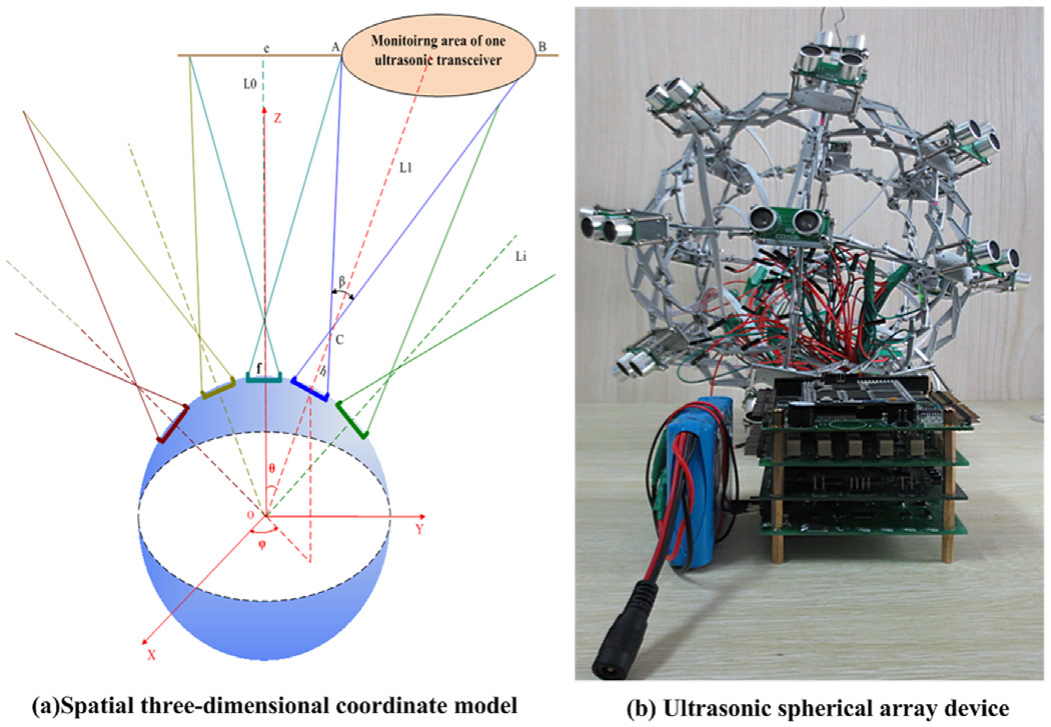
Ultrasonic omnidirectional sensor device.

This study establishes an ultrasonic spherical sensor device to detect omnidirectional deformation. The center of the sphere is considered the origin, and the ultrasonic transceiver arrays are localized on the surface of the sphere. A spatial 3D coordinate system is established, as shown in Fig 1(a). The ultrasonic spherical sensor array is used as a benchmark of spatial measurement to detect random structural deformation in underground space [26]. The designed device is shown in Fig 1(b).

The 3D space that is monitored through the ultrasonic array is organized and divided into a series of subspaces. These subspaces are distinguished from each other by identifying the ultrasonic sensors localized at the different latitude and longitude lines. The monitoring information for each direction in the 3D space is mapped into a latitude and longitude map to facilitate the visual management of the spatial monitoring information.

This study proposes a BHR composite network for visualizing deformation risk levels. The BHR composite network is composed of BPNN, Hopfield neural network, and RGB color space. The underground space deformation monitoring data are used as input in the BHR network to automatically classify and analyze the visualization of deformation risk levels.

### Part 1. BPNN Data Processing

The basic idea of the BPNN is to iteratively learn a certain number of samples, that is, inputs and expected outputs, until the error between the predicted and expected outputs satisfies the setting accuracy. In the BPNN, the signal propagates forward and the error propagates backward. The network includes input, hidden, and output layers. The output of a neuron j on the input and output layers is determined by Eq (1) [27]:

$$y_{t}{}^{}=f(\sum_{j=1}^{p}\nu_{jt}f(\sum_{i=1}^{n}\omega_{ij}e_{i}{}^{}-\theta_{j})-\lambda_{t}),\begin{array}{cc} & \left(i=1,2,\cdots ,n,j=1,2,\cdots ,p,t=1,2,\cdots ,q\right) \end{array}$$ (1)

where n, p, and qp represent the neuron number of the input, hidden, and output layers, respectively. The input sample component is represented by e_i_. The threshold value of the hidden layer is represented by *θ*_j_. The connection weight between the input and hidden layers is represented by *w*_ij_. The connection weight between the hidden and output layers is represented by *v*_jt_. The threshold value of the output layer is represented by *λ*_t_. The transfer function is represented by f.

The tan-sigmoid function is used as the BPNN transfer function, given that it can limit the output within the range of [-1, 1], as shown in Eq (2). After inputting the actual measured data into the trained BPNN, the stable weights and thresholds are acquired [27].

f(x)=tansig(x) (2)

### Part 2. Hopfield Network Data Processing

A type of feedback network, the Hopfield network has more than one stable state. It begins at a certain initial state and then reaches a stable state, which can be stored in the network by setting the network weight. Two types of Hopfield network exist: continuous and discrete network. Letting *u*_j_ and z_i_ represent the function input and output of neuron j at the moment of t, respectively, the input and output are determined through Eqs (3) and (4) [28, 29]

$$\mu_{j}=\sum_{i=1}^{q}\varphi_{ij}y_{i}-\delta_{j}$$ (3)

$$z_{j}=f(\mu_{j})=\{\begin{array}{c} 1\;\mu_{j}>0\\ -1\;\mu_{j}\leq 0 \end{array}$$ (4)

Where the threshold of neuron j is represented by *δ*_j_. The number of neurons is represented by q. The connection weight between neurons i and j is represented by φ_ij_. y_i_ The neuron input, that is, the actual output of the BPNN, is represented by yi.

The Hopfield network energy function is defined as [30]

$$E=-\frac{1}{2}\sum_{i=1}^{q}\sum_{j=1}^{q}\varphi_{ij}y_{i}z_{j}+\sum_{i=1}^{q}y_{i}\delta_{i}$$ (5)

When neuron j varies from time t to time t + 1, the energy variation of the neuron is as follows:

$$\Delta E_{j}=E_{j}(t+1)-E_{j}(t)=-\Delta z_{j}(\sum_{i=1}^{q}\varphi_{ij}y_{i}-\delta_{j})+\frac{1}{2}\Delta z_{j}\sum_{i=1}^{q}\varphi_{ij}y_{i}$$ (6)

When the state of neuron j changes, its energy variation is ΔE_j_≤0. Given that neuron j can be any one of the Hopfield network neurons, all the neurons of the network are in an updated state according to the same rules. Thus, the energy variation of the network should be Δ*E* ≤ 0.

The change in the network convergence involves an energy minimization process. Given that the energy function is bounded, the network reaches a steady state. This steady state is a discrete output of the Hopfield network, and the steady state condition is determined as follows:

$$z_{j}(t+1)=z_{j}(t)$$ (7)

Whether or not the network reaches a steady state is determined according to Eq (7). If the steady state or the training number satisfies the requirement, then the training is ended; otherwise, it should return to its former state and continue.

Given the aforementioned features of the Hopfield network, this study uses the discrete network. The designed stable states are used as the different risk levels of a sensor array. Through the Hopfield network, the data learned by the BPNN reach a stable state, which indicates that the deformation state is maintained at a certain risk level. The output value of the sensor node in the discrete Hopfield network is -1 or 1. The value is -1 when the neuron stays at an inhibitory state and the deformation is at a low risk level, and 1 when the neuron stays at an active state and the deformation is at a high risk level.

### Part 3. RGB Color Space

RGB color space is a 3D Cartesian space based on three variants: R, G, and B. The three vertices on the axis represent the three primary colors, namely, red, green, and blue. The different combinations of R, G, and B can form a total of about 16.78 million different colors. The different deformation risk levels can be distinguished by using the different colors formed by different combinations of RGB values.

### Part 4. BHR Network Data Processing

Each output data of the BPNN are at an equilibrium state. Each data converge into its own equilibrium state in the Hopfield-RGB network. The stable equilibrium values are then converted into RGB values. For example, if the discrete Hopfield neural network has three neurons, then the network output consists of three binary numbers; thus, eight stable equilibrium states at the most are present. These eight stable equilibrium states correspond to the eight vertices of the RGB color space. Using Eq (8), the output of the Hopfield network can be mapped into the RGB color space.

$$\{\begin{array}{c} R=\frac{255*(x_{1}+1)}{2}\\ G=\frac{255*(x_{2}+1)}{2}\\ B=\frac{255*(x_{3}+1)}{2} \end{array}$$ (8)

**Fig 2:**
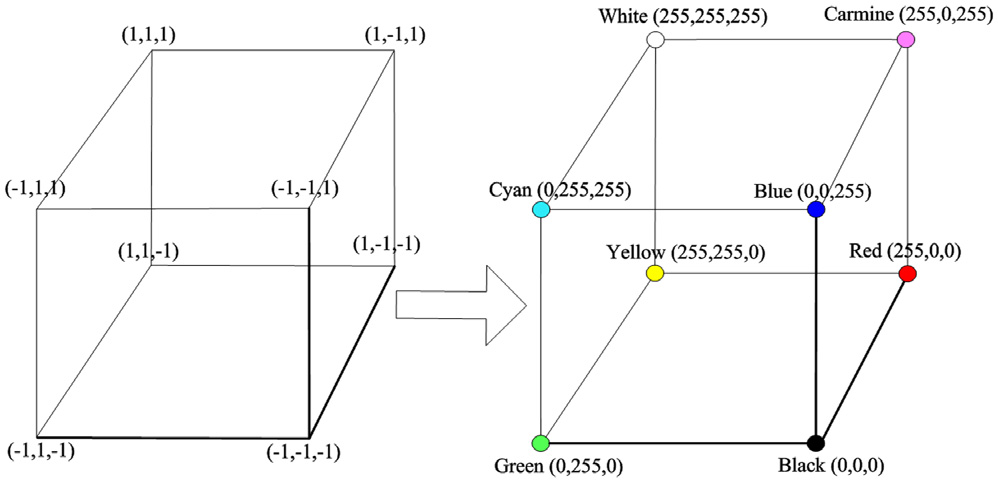
Hopfield-RGB network mapping model.

The equilibrium states in the Hopfield network are converted into certain colors and displayed in real time on the information structure unit of the longitude—latitude mapping model. The Hopfield-RGB network mapping model (with three neurons) is shown in Fig 2.

### Part 5. Visualization Model

**Fig 3:**
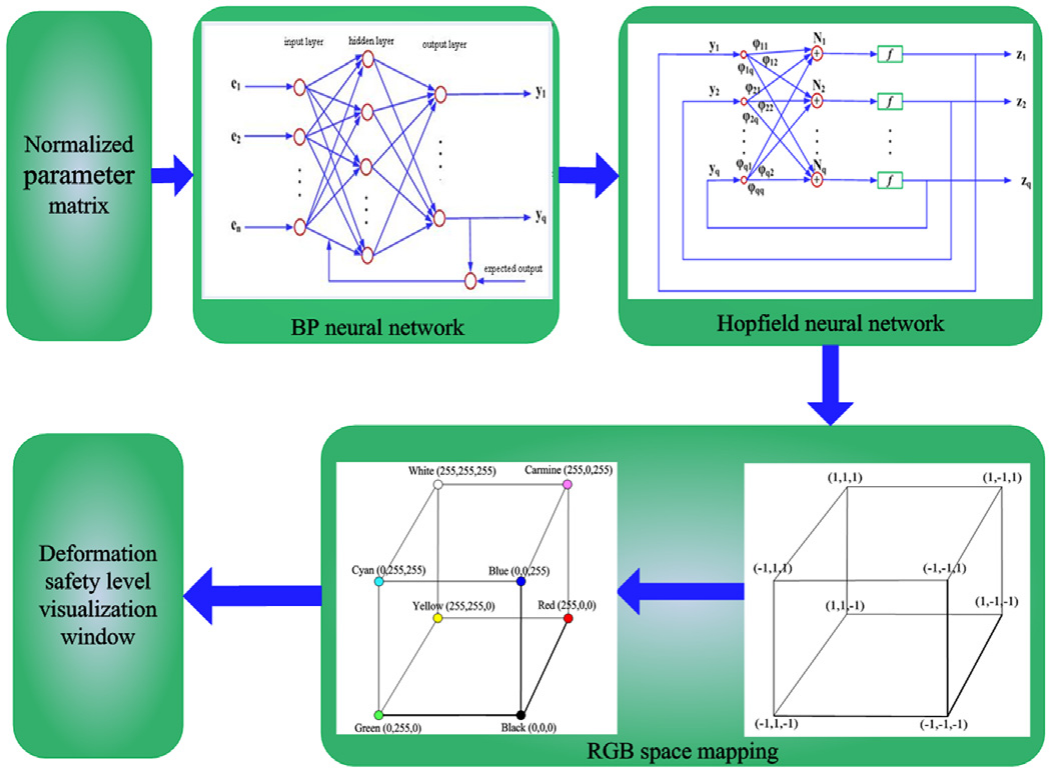
BHR network-based visualization processing model for deformation risk levels.

The deformation risk level is divided into N levels based on the deformation value and environmental parameters. A higher N corresponds to a higher risk level and therefore greater danger. The different risk levels of deformation are represented by different colors to distinguish among them. Based on the above discussion on the BHR composite network, a visualization processing model is proposed for deformation risk levels in underground space, as shown in Fig 3.

First, the real-time data monitored by the sensor array and environmental impact parameters are processed as a normalized parameter matrix, after which the matrix is inputted to the BPNN. Through the self-learning process of the BPNN, the stable weights and thresholds are acquired. Second, the Hopfield network is used to automatically classify the data learned by the BPNN. Finally, the deformation risk level classification is mapped into the color space and displayed in real time with different colors.

## Results and Discussion

### Model Experiment

The sensor nodes for the underground space deformation monitoring system are in different monitoring environments; thus, they have different measurement data and environmental parameters. This study considers three factors: object material, distance, and range difference.

1. Object material: Given that the monitoring object of the ultrasonic omnidirectional array is underground space, the properties of the monitoring object may be different for the monitoring area of each sensor node, which affects the deformation risk level. Setting the impact factor as a decimal value, the maximum impact factor of the different materials is 1, and the minimum is 0.
2. Distance: The distance between the monitored area and the sensor node affects the monitoring range and ultimately the deformation risk level. A greater distance corresponds to a higher deformation risk level. The maximum value of the parameter is set at 10,000 mm and the minimum value at 50 mm.
3. Ranging difference: The ranging difference directly reflects the deformation. A larger difference means a greater deformation. The corresponding monitoring region has a high deformation risk level. The maximum value of the parameter is set at 50 mm and the minimum value at 3 mm, that is, the value for the quantitative accuracy of the sensor.

### Visualization Experiment of a Single Sensor

**Fig 4:**
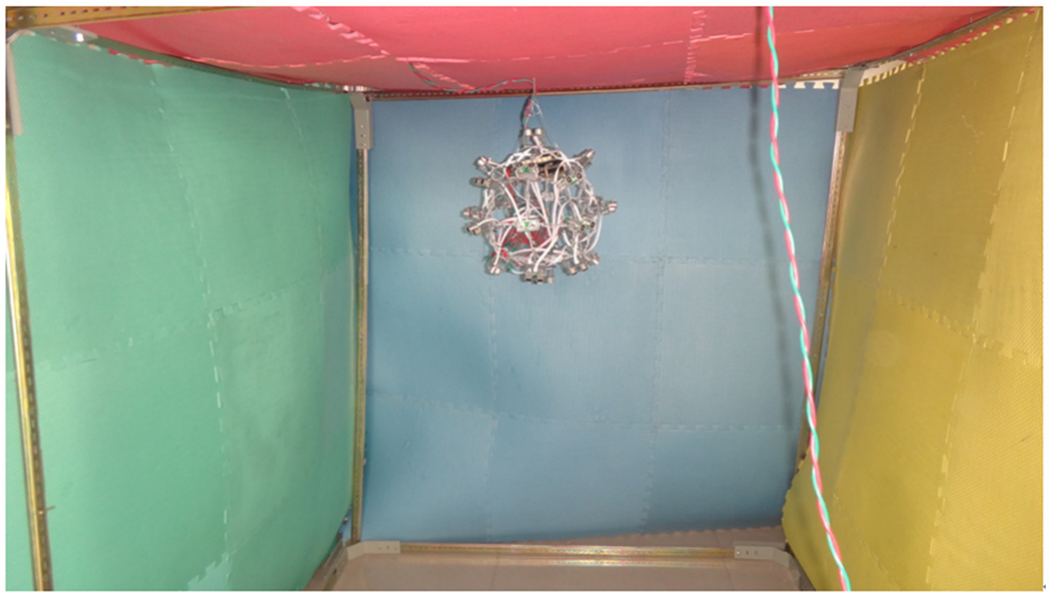
Experiment setup for acquiring the deformation risk level.

The methods can be used for deformation monitoring of an underground structure such as an artificial tunnel or a natural cave. However, applying real deformation to the monitored structure, which is in a stable state, is impossible. Therefore, the proposed method is illustrated via experiments in the laboratory. Considering the three factors above, an experiment was conducted, as shown in Fig 4. Elastic pads were used to build a semi-closed structure. The structural deformation was simulated by exerting force on the elastic pads.

First, the BPNN was trained using the data monitored by the sensor array and environmental impact parameters (the expected outputs are set as six risk levels). Then the structural deformation within the monitoring area of a single sensor node was measured. The pressure on the elastic deformable body was gradually increased, and the distances were acquired using the sensor. The deformation ranging differences were then acquired. Considering the parameter of the object material, a monitoring dataset was acquired as shown in Table 1.

**Table 1:** Deformation monitoring data of the sensor node located at longitude (E90) and latitude (0).

Number	Object material	Distance (cm)	Ranging difference(cm)	Number	Object material	Distance (cm)	Ranging difference(cm)
**1**	0.65	79.3	0	**11**	0.65	75.1	4.2
**2**	0.65	79.0	0.3	**12**	0.65	74.7	4.6
**3**	0.65	78.5	0.8	**13**	0.65	74.1	5.2
**4**	0.65	78.0	1.3	**14**	0.65	73.7	5.6
**5**	0.65	77.6	1.7	**15**	0.65	73.2	6.1
**6**	0.65	77.1	2.2	**16**	0.65	72.8	6.5
**7**	0.65	76.8	2.5	**17**	0.65	72.3	7.0
**8**	0.65	76.4	2.9	**18**	0.65	71.7	7.6
**9**	0.65	76.0	3.3	**19**	0.65	71.2	8.1
**10**	0.65	75.5	3.8	**20**	0.65	70.8	8.5

A total of 20 sets of data at different time points formed a parameter matrix, which was then normalized. The normalized data were used as input in the trained BPNN, and the data were converged to the range of [-1, 1]. The output results of the BPNN are shown in Table 2.

**Table 2:** Output results of the BP neutral network.

Number	y_1_	y_2_	y_3_	Number	y_1_	y_2_	y_3_
**1**	-0.9963	-0.0262	-0.5601	**11**	0.9282	0.5574	0.7865
**2**	-0.9947	-0.0433	-0.5431	**12**	0.9747	0.5270	0.8032
**3**	-0.9883	-0.0099	-0.4525	**13**	0.9944	0.4533	0.8081
**4**	-0.9687	0.1119	-0.2506	**14**	0.9978	0.3663	0.7890
**5**	-0.9225	0.2542	-0.0064	**15**	0.9992	0.1628	0.6992
**6**	-0.7512	0.4247	0.3169	**16**	0.9996	-0.1274	0.4766
**7**	-0.5284	0.4988	0.4707	**17**	0.9998	-0.6022	-0.2426
**8**	-0.0691	0.5583	0.6124	**18**	0.9999	-0.9269	-0.9239
**9**	0.4228	0.5815	0.6979	**19**	0.9999	-0.9845	-0.9931
**10**	0.8042	0.5771	0.7585	**20**	1.0000	-0.9944	-0.9987

**Fig 5:**
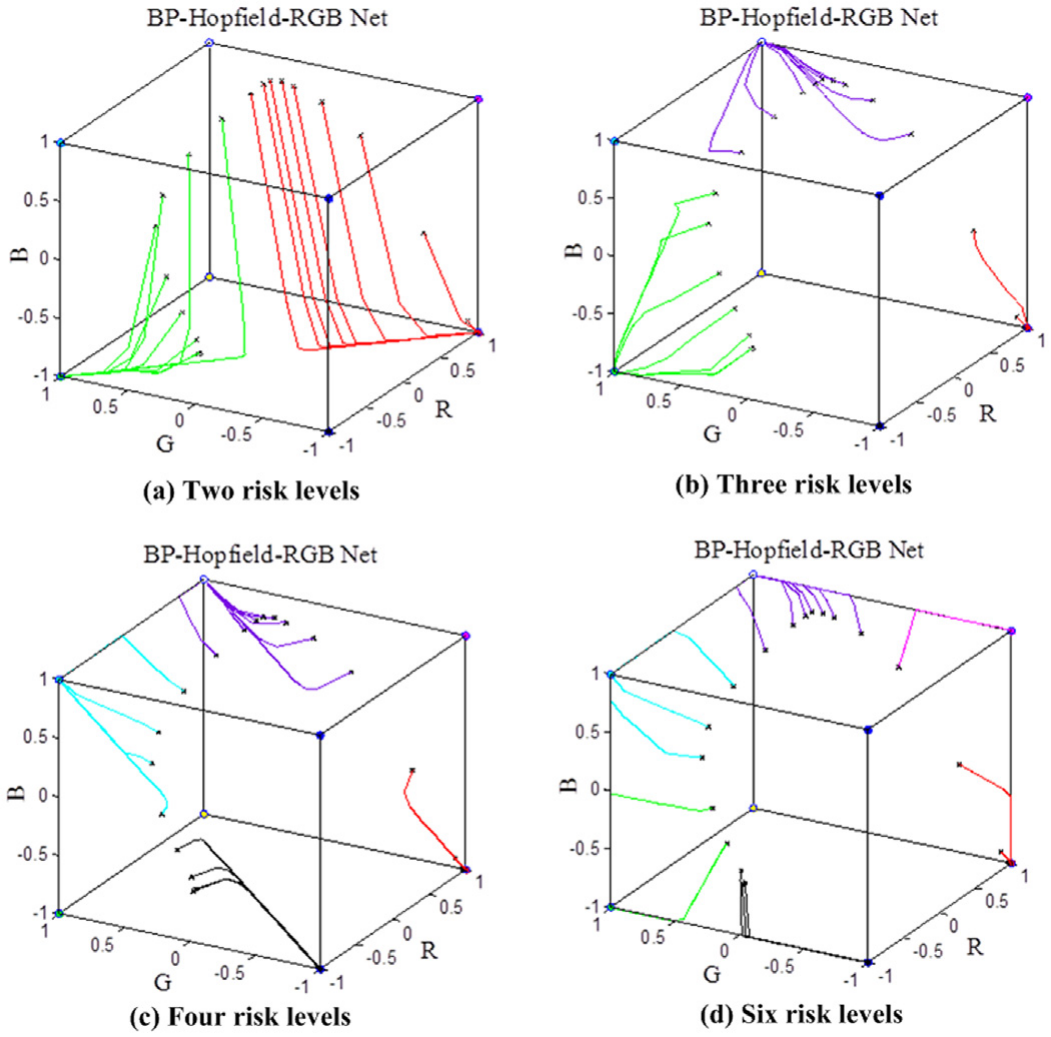
Experimental result graphs of different types of risk levels.

Second, the output matrix of the BPNN was used as input in the Hopfield-RGB network. Different stable equilibrium points were presented in the network, and the data of the matrix were automatically classified into different risk levels. The experimental results of different types of risk levels are shown in Fig 5. Fig 5(a) displays the result graph with two deformation risk levels; Fig 5(b), with three deformation risk levels; Fig 5(c), with four deformation risk levels; and Fig 5(d), with six deformation risk levels. The data processed by the BPNN converged into some stable equilibrium points along with the solid colored lines.

In Fig 5(a), the deformation risk levels were set to two. The stable equilibrium points of risk levels 2 to 1 were set to (1, -1, -1) and (-1, 1, -1), which correspond to red (the highest level) and green (the lowest level) in the color space, respectively. In Fig 5(b), the deformation risk levels were set to three. The stable equilibrium points of risk levels 3 to 1 were set to (1, -1, -1), (1, 1, 1), and (-1, 1, -1), which correspond to red (the highest level), white (replaced with purple in Fig 5 because white tracks cannot be seen clearly), and green (the lowest level) in the color space, respectively. In Fig 5(c), the deformation risk levels were set to four. The stable equilibrium points of risk levels 4 to 1 were set to (1, -1, -1), (-1, -1, -1), (1, 1, 1), and (-1, 1, 1), which correspond to red (the highest level), black, white (replaced with purple), and cyan (the lowest level) in the color space, respectively. In Fig 5(d), the deformation risk levels were set to six. The stable equilibrium points of risk levels 6 to 1 were set to (1, -1, -1), (1, -1, 1), (-1, -1, -1), (1, 1, 1), (-1, -1, 1), (-1, 1, -1), and (-1, 1, 1), which correspond to red (the highest level), carmine, black, white (replaced with purple), green, and cyan (the lowest level) in the color space, respectively.

### Data Visualization Experiment of Omnidirectional Sensor Array

**Fig 6:**
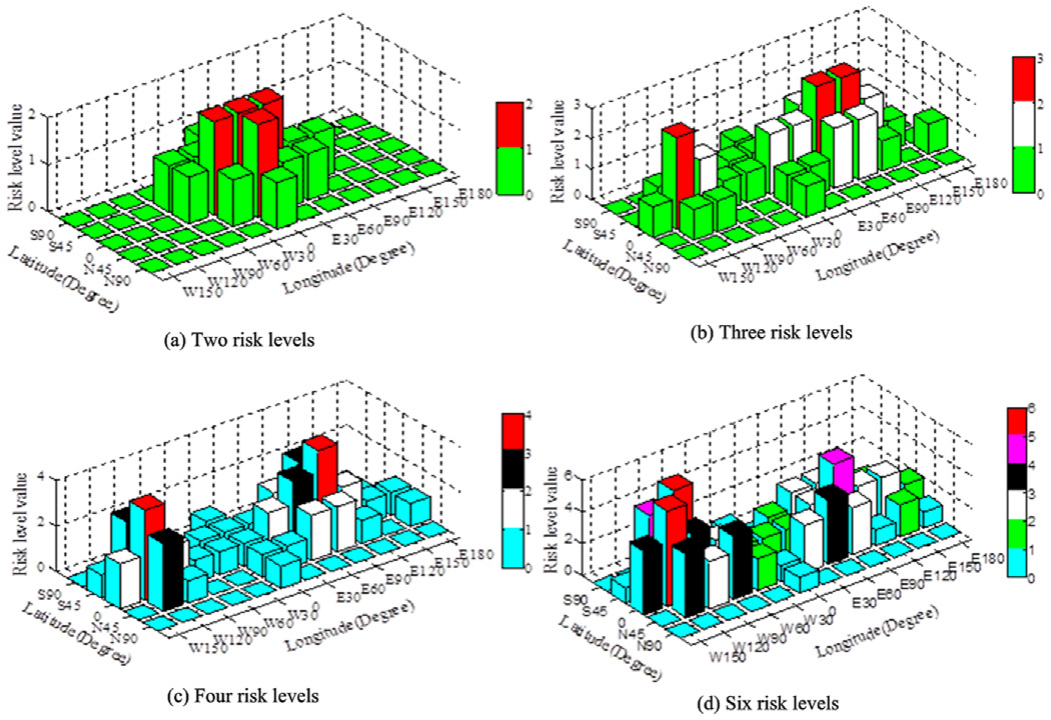
Visualization results of different types of deformation risk levels.

Different pressures were exerted on different areas on the elastic pads to visualize the sensor array monitoring data. The deformation data monitored by the sensor array were used as input in the trained visualization model of the deformation risk level. The visualization results are shown in Fig 6; the risk levels are divided into two, three, four, and six levels in Fig 6(a), 6(b), 6(c), and 6(d), respectively. The horizontal coordinates represent the latitude and longitude of the earth’s longitude—latitude mapping model, which were used to locate the sensor nodes. The dangerous zones were located afterward; different colors were used to represent different risk levels. The sensor array monitoring data were visualized, and the risk level values were displayed synchronously.

### Comparison with Other Algorithms

#### Part 1. Dataset introduction

A standard dataset was used to compare the chosen algorithms. Different algorithms were used to process the dataset to select the suitable classification algorithm.

Given that this study focuses on underground space monitoring, a benchmark dataset for seismic bumps was selected [18]. Mining activities are always related to the occurrence of various forms of danger, which are commonly called mining hazards. The dataset described the problem of high-energy (higher than 10^4^ J) seismic bump forecasting in a coal mine, and the data were obtained from two longwalls in a Polish coal mine. The dataset was a matrix with 2584 instances and 19 attributes. The present study selected three attributes, namely, genergy, gdenergy, and gdpuls, including a total of 1760 instances, in which 1600 instances were used for network training and 160 instances for network testing. The test results were then compared. The seismoacoustic attribute was also selected to calculate the accuracy rate of the classifier. The four selected attributes are described as follows:

1. Genergy: the seismic energy recorded in the previous shift by the most active geophone (GMax) out of all the geophones that monitor the longwall.
2. Gdenergy: a deviation of energy recorded within the previous shift by GMax from the average energy recorded in the eight previous shifts.
3. Gdpuls: a deviation of a number of pulses recorded in the previous shift by GMax from the average number of pulses recorded in the eight previous shifts.
4. Seismoacoustic: the result of the shift seismic hazard assessment in the mine obtained through the seismoacoustic method.

#### Part 2. Comparison of the pre-processing algorithms

This study normalized the environmental impact parameters and the data monitored by ultrasonic omnidirectional sensors, which then formed a parameter matrix. Before it was classified, the parameter matrix was pre-processed, and a pretreatment algorithm was used to converge all the parameters into the range of [-1, 1]. Therefore, three classic algorithms—the BPNN, radial basis function neural network (RBFNN), and generalization regression neural network (GRNN)—were selected to pre-process the seismic bump dataset.

BPNN is known as the error backpropagation neural network, which is a typical multilayer forward neural network. In the network, the signal forwards transmission and the error backs propagation. Unlike that of the global network, the action function of the RBFNN is localized and can approximate any nonlinear function. The GRNN is a one-passing learning algorithm, which approximates any arbitrary function between the input and output vectors, thereby directly drawing the function estimation from the training data [31].

**Fig 7:**
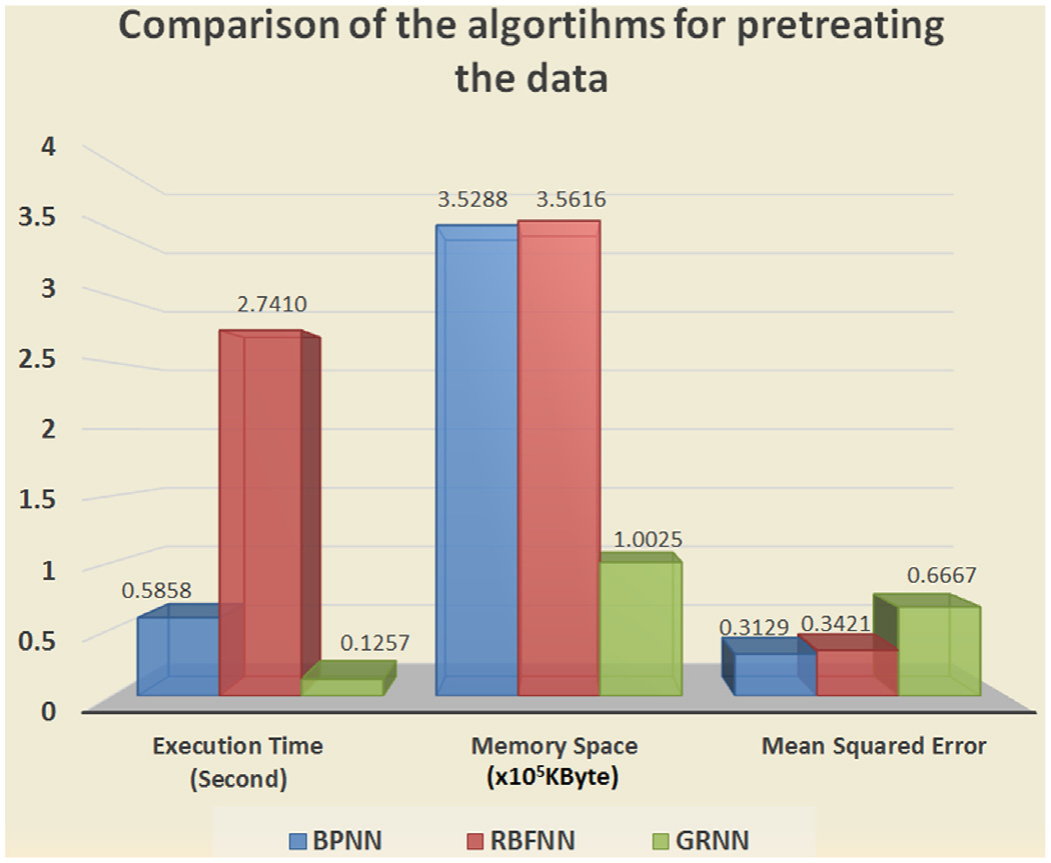
Comparison of the algorithms for pretreating the data.

The experiment was conducted under the same conditions. The experimental hardware platform was Intel(R) Pentium(R) CPU 2.6 GHz (two CPUs) 4 GB RAM, and the software experimental platform was Microsoft Windows XP, C language. The three algorithms above were used to process the standard dataset. The compared items were execution time, running memory space, and mean squared error; the results are shown in Fig 7.

**Fig 8:**
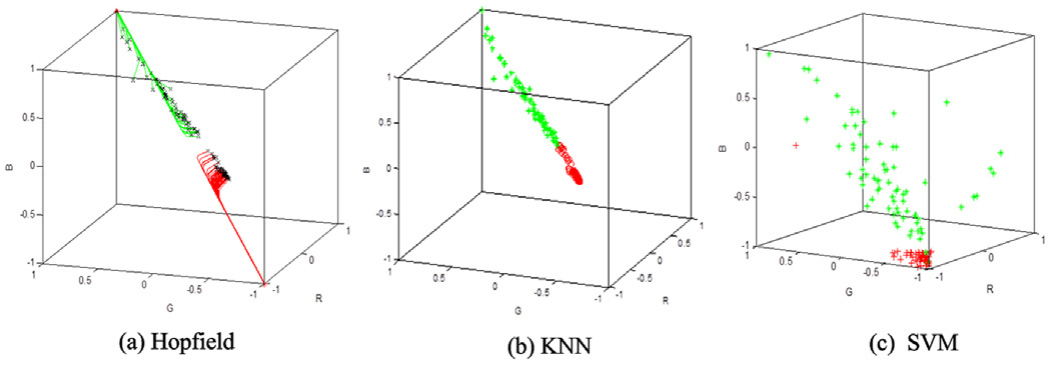
Classification results of the three algorithms.

The comparison of the BPNN and RBFNN in Fig 8 shows that the mean square error and the memory of the running space have little difference. However, the BPNN uses less time than the RBFNN. The most important factor is that the BPNN also has a smaller mean squared error than the RBFNN and GRNN. Given that it integrates the performance of the three algorithms, BPNN is considered the most suitable algorithm for preprocessing data in this study.

#### Part 3. Classification algorithm comparison

Three classic algorithms—the Hopfield neural network, k-nearest neighbor (KNN), and support vector machine (SVM)—were selected to classify the 160 sets of preprocessed data. The algorithms were evaluated in terms of execution time, running memory space, and classification accuracy. The classification results are shown in Fig 8.

**Fig 9:**
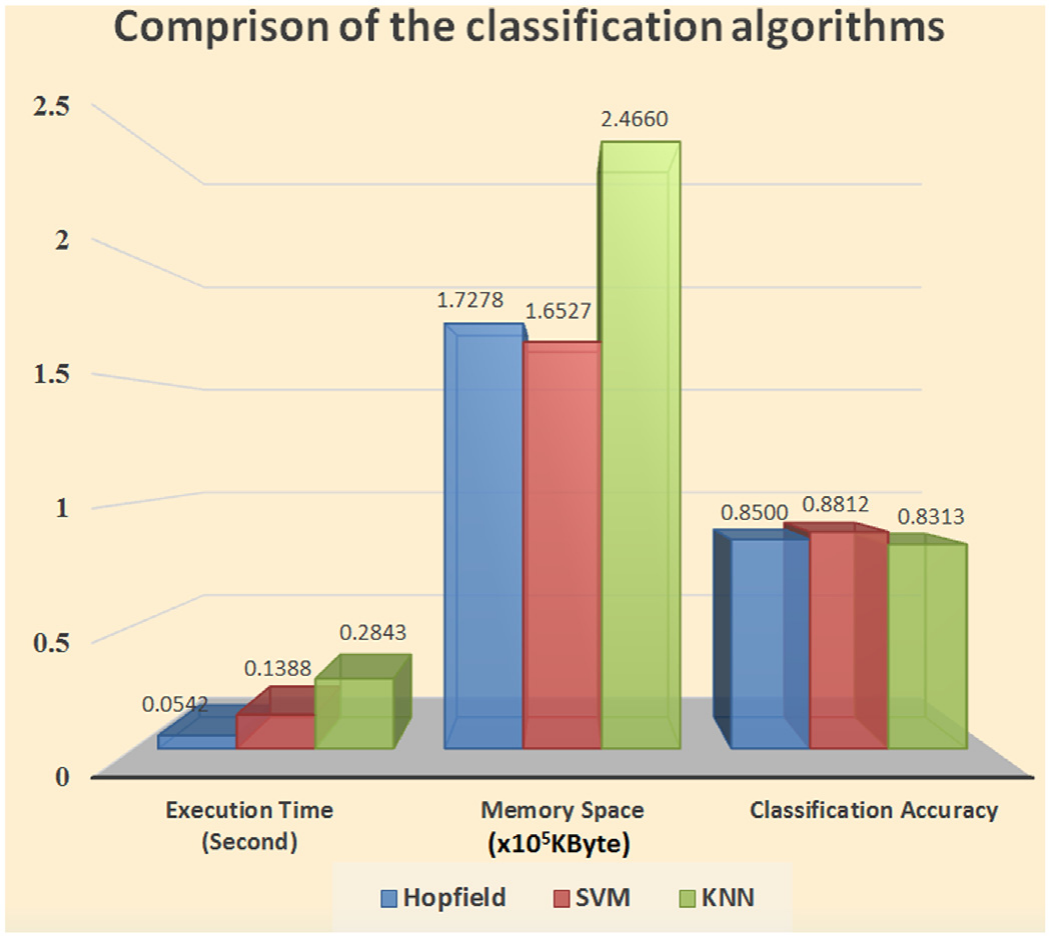
Comparison of the classification algorithms.

The data are classified into two types, as shown in Fig 8. The green part represents seismoacoustic = 1, which indicates “lack of hazard.” The red part represents seismoacoustic = 2, which indicates “hazard.” The result of the Hopfield neural network is more concise and has better classification performance than other algorithms. A detailed comparison of the results of the three algorithms is shown in Fig 9.

The comparison of the Hopfield neural network and SVM algorithm shown in Fig 9 shows that the two algorithms have little difference in terms of running space memory and classification accuracy. However, the Hopfield network uses less time than the SVM algorithm. A comparison of the Hopfield neural network and KNN algorithm shows that the former has higher classification accuracy and uses less time than the latter. Given that it integrates the performance of the three algorithms, the Hopfield neural network is considered the most suitable classification algorithm in this study.

### Comparison with Existing Techniques

The visualization technologies for safety monitoring are closely connected to measurement systems. Thus, the proposed method is compared with data processing systems on the basis of three techniques, namely, photogrammetric, total station, and 3D laser scanning techniques. These techniques have been widely used for deformation monitoring for the past decade. Specifically, photogrammetric technique places targets on the gallery vault. Useful information is then obtained from photographs captured by an optical camera [19]. In total station technique, an electronic theodolite is integrated with an electronic distance meter to read slope distances from the instrument to a particular point. Distance is measured by a modulated infrared carrier signal that is generated by a small, solid-state emitter within the optical path of the instrument and is reflected by either a prism reflector or the object under survey [20]. In 3D laser scanning technique, a 3D laser scanner is employed to scan the surface of the target object to obtain point clouds of either thousands or millions of coordinates with millimeter accuracy. The 3D profile can be constructed via data merging [6]. Performance parameters considered for comparison include accuracy, distance, display speed, anti-dusting capability, the need for manual assistance, capability to handle environmental parameters, adaptability in topography monitoring, and visualization results (Table 3).

**Table 3:** Comparison of visualization techniques on the basis of typical measurement systems.

Characteristics	BHR composite network visualization method based on ultrasonic technique	Data processing system based on photogrammetric technique	Data processing system based on total station technique	Data processing system based on 3D laser scanning technique
**Accuracy**	1.5 mm	0.2 mm	1 mm	2 mm
**Distance**	Near (< 10 m)	Near (< 10 m)	Far (>1000 m)	Far (>1000 m)
**Display speed**	Quick (< 10 s)	Quick (< 10 s)	Slow (> 10 s)	Slow (> 60 s)
**Anti-dusting capability**	Yes (Ultrasound is insensitive to dust)	No (Photographic lens should be kept clean)	No (Prism reflector should be kept clean)	No (Laser head should be kept clean)
**Need for manual assistance**	No	Yes	Yes	No
**Capability to handle environmental parameters**	All environmental parameters can be considered in the model.	Only 3D coordinate data for multiple points are considered. Other environmental factors are neglected.	Only 3D coordinate data for a single point are considered. Other environmental factors are neglected.	Only point cloud data are considered. Other environmental factors are neglected.
**Adaptability in topography monitoring**	Universal—for all kinds of topography	The topography must be illuminated.	The topography must be illuminated.	Universal—for all kinds of topography
**Visualization results**	Profile data and risk levels can be displayed to help locate the danger point.	Software such as OpenGL is used to construct the 3D profile. Risk levels must be displayed via an additional mechanism.	Professional software such as Spectra Precision Survey Pro is used to satisfy the need for surveys. Risk levels must be displayed via an additional mechanism.	Fine 3D construction profiles can be produced. Risk levels must be displayed via an additional mechanism.

The comparison indicates that although the accuracy and distance of the proposed method are not ideal, this method has significant potential for use in the monitoring of structural safety given its quick display speed, anti-dusting capability, the lack of a need for manual assistance, capability to consider environmental parameters, adaptability in topography monitoring, and capability to display risk levels.

## Conclusion

This study investigated the visualization model of the deformation risk levels of the ultrasonic omnidirectional array. Multiple environmental parameters were considered. The BPNN and Hopfield neural network were adopted for pre-processing and classifying the sensor data. The data processing results were mapped into RGB color space, which visualized the deformation risk levels of the sensor array. The visualization results facilitate the determination of the presence and location of danger. Experiments and comparison with other algorithms demonstrate that the method is characterized by intelligent, dynamic, and real-time features and can therefore be used as a universal model for safety monitoring in underground space.
